# A Case of Hydrocephalus Internus Cured by Mercury; with Some Remarks on That Disease

**Published:** 1781-06

**Authors:** 


### SECTION II.

### ESSAYS AND OBSERVATIONS.

I. *A Case of Hydrocephalus internus cured by mercury; with some remarks on that disease. By* Thomas Aery, *sen. M. D. Physician at Whitehaven. Read* May 20, 1781.

JANUARY 4th 1780, I was called to Master Spittall of this town, aged nine years, of rather a pale complexion and relaxed habit. He was seized a week before this with acute pain of the whole head, the teguments of which were sore to the touch. At the time I saw him he complained of nausea, anorexia, erratic pains of the extremities and head; and of thirst especially towards evening. His tongue was clean, his pulse rather frequent. I began by prescribing an emetic, and afterwards emptied the bowels by a gentle purge, at the same time advising the application of blisters behind the ears. The sickness left him after taking the emetic, but the other symptoms remained. After this he took at different times the saline mixture, the bark, and other remedies, but without any apparent effect. The discharge from the blisters was likewise continued.

Jan.

Jan. 20th. He complained that the light was painful to his eyes, the pupils of which were much dilated; he was troubled with ſtrabiſmus, and could not diſtinguiſh capital letters in print; had double viſion; ſpoke unuſually ſlow; was affected with ſtupor, and could not riſe out of bed. His countenance, however, was not altered, but his extremities were conſiderably emaciated. His appetite continued good; he ſlept well, had but little thirſt, his abdomen was rather tenſe and hard, but his ſtools and pulſe were regular.

24th. The ſymptoms were much the ſame.—Mr. Hamilton, an ingenious ſurgeon and apothecary who attended the patient with me, was of the ſame opinion as myſelf concerning the nature of the diſeaſe which we aſcribed to water in the brain; upon which I put him under a courſe of mercury as recommended by the ingenious Dr. Dobſon and Dr. Percival in the Edinburgh *Med. Com.* Vol. 5. p. 174. & Vol. 6. p. 219. I was the more readily induced to try this method, from my having many years ago found mercury uſeful when given as an aperient and deobſtruent in caſes of dropſy.

Feb. 1. He began by taking from one to two grains of calomel twice a day. Mercurial ointment was likewiſe rubbed into his legs every night.

Feb. 10th. The pain of his head was abated.

11th. A vomiting came on laſt night, but was ſoon removed by his taking the ſaline mixture with tinctura thebaica. He now complained of his tongue being very ſore. I found it with a little apthous redneſs, but no appearance of ſalivation.

22d. He ſaid he was better for the firſt time.

24th. He ſat up, and could read ſmall print. After this he continued the uſe of mercury in ſmall doſes till the 4th of March, when all the ſymptoms being removed, he diſcontinued it, and has continued well to the preſent time.

From Feb. 4th to March 8th, he took forty-two grains of calomel, and rubbed in three ounces and upwards of *ung. merc. fort.* without producing any ſalivation, or any apparent evacuations, except the night that he puked. In Dr. Dobſon's and Dr. Percival's caſes the mercury ſalivated.

It is a well known fact, which I can atteſt from repeated experience, that in curing the lues venerea a ſalivary diſcharge is not abſolutely neceſſary to exterminate the venereal virus, for I have found ſome patients in whom a ſalivation could could not be eaſily raiſed (as in this caſe) and many inſtances where the diſcharge could not be procured, though a mercurial courſe had been regularly conducted for this purpoſe; and yet a cure was obtained, although a greater quantity of mercury was required for that purpoſe. This likewiſe was the caſe in our patient with the hydrocephalus internus.

It may be preſumed, that in near forty years that I have been engaged in the practice of phyſic, I muſt have ſeen ſeveral patients in this diſeaſe, yet of all that I have attended, I never ſucceeded in any but the preſent caſe. It is true I never gave mercury but to one other patient ſome years before, and then in ſmall doſes, and without any intention to ſalivate; and from mercury given in ſo ſmall a quantity the ſame ſucceſs could not be expected. In the Edinburgh *Med. Comment.* I find the hiſtories of ſeven patients in this diſorder treated with mercury, of whom five were cured, and one relieved by it. In the *Med. Obſ. and Inq.* Vol. 4. we meet with one inſtance of recovery from the ſame complaint, which makes ſix cured out of eight, a great proportion cured of ſo fatal a diſeaſe.

It is not improbable that ſeveral more patients may have been treated by different practitioners with mercury, ſince Dr. Dobſon firſt recommended it, than we are acquainted with, and unſucceſsfully, as phyſicians in general are not over-fond of publiſhing their want of ſucceſs. Why may not mercury ſometimes cure this diſorder without ptyaliſm, as well as it does ſometimes the lues venerea? And may not mercury act in this caſe as a deobſtruent, by removing the obſtructions in the ſyſtem of abſorbents in the ventricles of the brain, and rendering them capable again of abſorbing the extravaſated fluid, and ſo cure the diſeaſe?

*Whitehaven,*
*May* 21, 1781.

